# Hospitals, Tenders, and Contractors

**Published:** 1897-03-13

**Authors:** 


### HOSPITALS, TENDERS, AND CONTRACTORS.

The articles which have appeared in The Hospital on Hospital Commissariat have attracted a great deal of attention to the subject of tenders and the best and most economical method of securing supplies for hospitals and kindred institutions. In "The Uniform System of Accounts, Audit, and Tenders" (London, the Scientific Press), a complete set of forms of tender and other particulars are given, which may usefully be consulted both by secretaries and contractors. Anyone who masters them will be able to understand the best means of securing the supply of goods of uniformly average quality and excellence. Most hospitals have their own system of supply. Tenders are usually issued half-yearly, in some cases annually; but to make this system work effectually it is desirable that one or more persons in authority should make themselves familiar with the state of the markets, and that those who are responsible for the acceptance of the goods should be capable of preventing the introduction of inferior articles at any time throughout the period of contract. A certain number of firms make it their business to tender for the supply of institutions. They and the staff they employ have now so exact a knowledge of the requirements and conditions that no excuse is possible where inferior goods are tendered for acceptance. There can be no doubt that both institutions and contractors would be helped considerably by a tacit arrangement to advertise both tenders and supplies in the same journal, and to send to that journal for publication the tenders which may be accepted, and the prices fixed. In this way new contractors—and competition in these matters is highly desirable—would have an opportunity of bringing their improved methods of supply to the notice of those who are likely to benefit most by their adoption, and institutions would be able to protect themselves against the amazing differences which are sometimes exhibited by the prices paid for apparently the same article by different institutions under the contract system. We have often urged that each Board or Committee should appoint a small number of its members to investigate the contracts and to exercise a watchful eye upon the way in which each contractor does his work. Important as the form of contract undoubtedly is, the results attained would be materially improved if every institution were to have its Contract Committee, and if both institutions and contractors were to agree to insert their advertisements and the results of the competition in regard to contracts in the same newspaper. The Hospital now circulates throughout every medical institution in this country, as well as in India, the British Colonies and abroad, and we shall be very happy to place our columns at the disposal of hospital managers and contractors, if the ideas here suggested commend themselves to their adoption.

